# Assessing the Efficacy of Hyperbaric Oxygen Therapy on Facelift Outcomes: A Case–Control Study Comparing Outcomes in Patients With and Without Hyperbaric Oxygen Therapy

**DOI:** 10.1093/asjof/ojad065

**Published:** 2023-07-14

**Authors:** Omar Fouda Neel, Ahmed Hafez Mousa, Reem Abdulmonem Al-Terkawi, Moamen M Bakr, Hatan Mortada

## Abstract

**Background:**

Wound healing remains among the most concerning complications in aesthetic surgery. The use of hyperbaric oxygen therapy (HBOT) is an accepted method of supporting wound healing.

**Objectives:**

The aim of this study is to assess the role of HBOT in postoperative healing and complication rates following facelift surgery.

**Methods:**

This case–control study comprised facelift patients who received HBOT and those who did not between 2019 and 2022. Data were extracted from the patients’ medical records, with the primary outcomes being the presence of complications, wound-healing duration, and patient satisfaction.

**Results:**

The authors recruited 20 female patients who underwent facelift for this study, with 9 patients in the HBOT group and 11 patients in the control group. The average number of HBOT sessions received was 7.22, and each session lasted an average of 78 ± 5 min. The duration of wound healing in the HBOT group ranged from 7 to 30 days (mean of 13.3 days), whereas the control group ranged from 6 to 90 days (mean of 36.9 days). This indicates a statistically significant shorter time to wound healing in the HBOT group compared to the control group (*P* < .001).

**Conclusions:**

Future prospective randomized controlled trials with larger sample sizes and blinding are needed to further evaluate the potential benefits of HBOT in the postoperative period. Nonetheless, our findings suggest that HBOT may be a promising adjunctive therapy for patients undergoing facelift surgery.

**Level of Evidence: 3:**

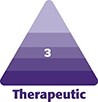

Aesthetic surgery has gained increasing popularity over the past few decades, and with the rise in demand, there has been a corresponding increase in the number of surgical procedures performed.^1^ However, like any surgical procedure, aesthetic surgery carries the risk of complications, including wound-healing problems.^1,2^ Various methods have been utilized in attempts to manage wound-healing problems. The use of hyperbaric oxygen therapy (HBOT) is an accepted method of supporting wound healing.^3^ HBOT serves as “primary” or “adjunctive” therapy for a wide range of pathologies.^4^ HBOT dates back to the 1600s when Dr Henshaw built the first hyperbaric chamber.^5,6^ HBOT involves breathing 100% oxygen while being placed in a hyperbaric chamber with increased atmospheric pressure. The theory behind HBOT is that it increases the oxygen supply to tissues, promotes tissue repair and regeneration, reduces swelling and inflammation, and enhances the immune system’s ability to fight infection. These factors are all essential to the healing process following surgery.^6–8^ Tissue hypoxia triggers the cellular inflammatory cascade and initiates the wound-healing process, and it is crucial to maintain a minimum oxygen pressure of 30 mm Hg in tissues to create a suitable environment for wound healing. HBOT is a safe and effective treatment option that promotes the systematic repair of ischemic tissues by enabling the direct diffusion of oxygen through the inhalation of 100% oxygen in a pressurized chamber. There have been numerous instances in plastic surgery in which HBOT has proven to be beneficial, including the treatment of postfiller necrosis, vascular occlusion, crush injuries, ulcers that are difficult to heal, hand replantation, and deep skin infections.^8^

This study aimed to evaluate the effectiveness of HBOT in improving outcomes in patients undergoing facelift surgery based on a single surgeon’s experience. Patients who received HBOT were compared with those who did not, and the effects of HBOT on wound healing, complications, and patient satisfaction were assessed.

## METHODS

### Study Design and Patient Selection

**Figure 1.**
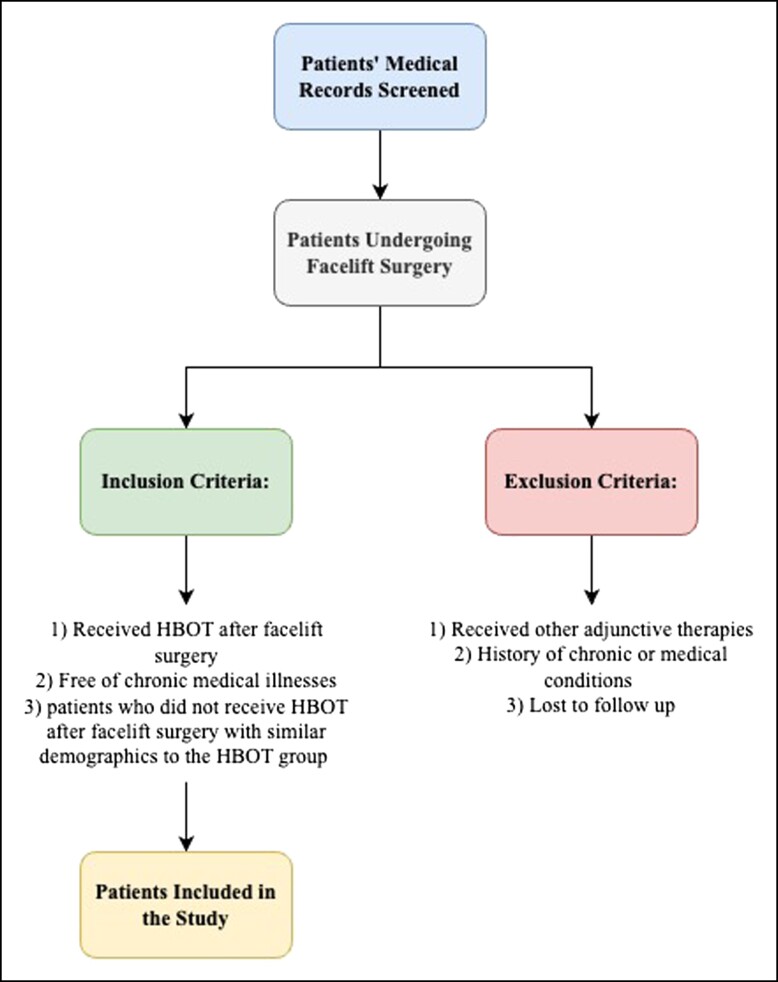
Flow chart of patient selection: inclusion and exclusion criteria for a study on HBOT following facelift surgery.

This study was a case–control study that compared the postoperative healing and complication rates between patients who underwent HBOT and those who did not receive HBOT after facelift surgery, which was conducted based on a single surgeon’s experience in a private practice plastic surgery center in Riyadh, Saudi Arabia. A list of patients who met the inclusion criteria was compiled. The study included patients who underwent aesthetic surgery performed by the senior author (O.F.N.) between January 1, 2019 and December 31, 2022. Patients with missing data were excluded. The inclusion criteria were as follows: (1) patients who received HBOT as an adjunctive therapy after facelift surgery (HBOT group), and (2) patients who did not receive HBOT after facelift surgery with similar demographics to the HBOT group (control group). The exclusion criteria were as follows: (1) patients who received other adjunctive therapies (eg, platelet-rich plasma) after aesthetic surgery, (2) patients with a history of chronic wounds or medical conditions that affect wound healing, and (3) patients who were lost to follow-up (Figure 1). Patient selection in the cohort group was based on specific criteria, including age, gender, medical background, and preoperative characteristics. These criteria were chosen to minimize potential confounding variables and enhance comparability between the groups. The decision to include HBOT in the study was driven by the senior author’s recent incorporation of HBOT after facelift surgeries. This study was initiated to compare outcomes before and after the implementation of HBOT in the senior author’s practice.

### Data Collection

After conducting a review of the literature with similar objectives, a data collection sheet was developed, which contained a range of variables for data collection in this retrospective study. Patient data were extracted from the medical record database using an excel sheet. The collected data included demographic information (age, sex, body mass index [BMI]), medical history, preoperative hemoglobin, operative time in minutes, and length of hospital stay. For patients who received HBOT, the number of sessions, indications for the therapy, HBOT protocol, duration of sessions, time until the wound healed in days, and whether the HBOT was administered before or after the surgery were recorded. Patients in the HBOT group were asked about their satisfaction and experience with the treatment. The control group received standard postoperative care without HBOT. In our study, the assessment of wound-healing duration was based on close observation and patient reports. Patients were asked to provide updates on the progress of wound healing until complete healing was achieved. In addition, all patients underwent a consistent facelift technique, which involved a deep plane facelift approach with a modified facelift incision. The specific operative details, including the SMAS technique and treatments for the neck, were standardized across all patients. It is important to note that no drain was placed in any of the patients, and the postoperative dressing consisted of gauze dressing with antibiotic ointment. Patients were discharged on Day 0 postoperative with prescribed analgesia and antibiotics.

### Ethical Approval

Following ethical approval from the Institutional Review Board at King Saud University Medical City, King Saud University, Riyadh, Saudi Arabia (Ref. No. 24/0167/IRB) and in accordance with the Declaration of Helsinki, patient medical records were collected for this case–control study. The STROBE checklist was utilized to guide the conduct and reporting of the study,^9^ and all methods were carried out in compliance with the relevant guidelines and regulations. Written consent was provided, by which the patients agreed to the use and analysis of their data.

### Statistical Analysis

Data were analyzed using descriptive statistics (mean, standard deviation) for continuous variables and frequency distributions for categorical variables. The χ^2^ test and Fisher’s exact test were used to compare the time until the wound healed between the HBOT group and the control group. A *P*-value of <.05 was considered statistically significant. Multivariate logistic regression analysis was performed to identify the independent predictors of postoperative complications. All statistical analyses were performed using SPSS version 24 (IBM Corp., Armonk, NY).

## RESULTS

Our study enrolled a total of 20 female patients who underwent facelift surgery, with 9 patients in the HBOT group and 11 patients in the control group. The patients had a mean age of 50.5 years (ranging from 28 to 65 years old), and only 1 patient in the control group was a smoker. The average BMI was 26.49 kg/m^2^, with a range of 20.31 to 35.79 kg/m^2^. Among the patients, 11 had no chronic illnesses, while 4 had thyroid-related disorders, 1 was diabetic, 2 had asthma, and 1 had hypertension and depression. All patients underwent their surgeries as outpatient procedures and were discharged on the same day. Table 1 provides an overview of the demographic characteristics of the patients included in the study.

**Table 1.** Demographic Characteristics of Patients Under Study

Parameter	Category	Overall, *N* = 20	Received HBOT		
			Yes, *N* = 9	No, *N* = 11	*P*-value
Age	Year (mean ± SD)	50.05 ± 13.2	54.7 ± 12.1	46.18 ± 13.7	.385
Sex	Female	20 (100%)	9 (45%)	11 (55%)	
Married	Yes	17 (85%)	8 (47%)	9 (52.9%)	.254
Children	Yes	15 (75%)	6 (40%)	9 (60%)	.436
Work	Yes	5 (25%)	2 (40%)	3 (60%)	.795
Smoking	Yes	1 (10%)	0 (0%)	1 (9%)	.353
BMI	kg/m^2^ (mean ± SD)	26.49 ± 5.5	28.48 ± 4.3	24.85 ± 4.2	.395
OR time	Minutes (mean ± SD)	175.5 ± 24.9	173.3 ± 36	177.2 ± 39	.766
Time until the wound healed	Days (mean ± SD)	26.3 ± 24.9	13.3 ± 6.9	36.91 ± 29.5	<.001

BMI, body mass index; HBOT, hyperbaric oxygen therapy; OR, operative; SD, standard deviation.

Most of the 9 patients who received HBOT were treated to promote general wound healing, and 1 patient received HBOT due to wound-healing-related complications (*n* = 1). The average number of HBOT sessions received by patients was 7.22, with a range of 1 to 15 sessions. The first HBOT session was initiated within 24 h of surgery. Each HBOT session lasted an average of 78 ± 5 min, ranging from 60 to 90 min, and the pressure used during HBOT was standard at 2.0 ± 0.1 ATA. There was no significant difference in HBOT sessions, timing, duration, or pressure used between the facelift subgroups. All patients were exposed to 100% pure oxygen in a hyperbaric chamber during HBOT.

**Figure 2.**
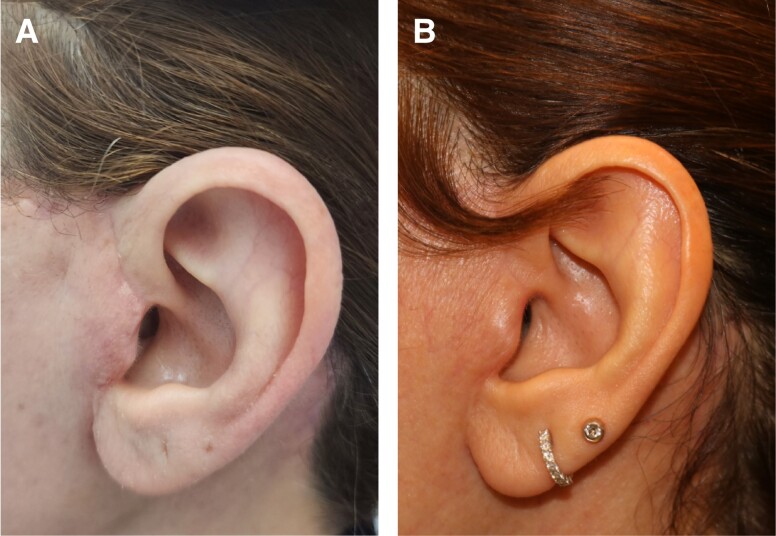
A depiction of the improvement of a 42-year-old female who underwent a facelift and experienced delayed wound healing, and hence underwent 5 sessions of HBOT. The patient is shown (A) a few days after the facelift surgery, prior to receiving HBOT and (B) after completing 5 sessions of HBOT.

The time until wound healing in the HBOT group ranged from 7 to 30 days (mean of 13.3 days), compared to the control group, which ranged from 6 to 90 days (mean of 36.9 days), indicating a statistically significant shorter duration of wound healing in patients who received HBOT (*P* < .001). Among the 9 patients who received HBOT, 6 of them agreed that it was important and helped in their wound healing when asked if they would recommend HBOT after surgery (Table 2). No complications, including barotrauma requiring ear tube placement, were observed in the HBOT treatment group during the study period. The study’s average follow-up time was 9 months, ranging from 4 to 15 months. Figure 2 illustrates a 42-year-old facelift patient’s progress with delayed wound healing. Image A is pre-HBOT, and Image B shows her condition after 5 HBOT sessions.

**Table 2.** Variables Related to HBOT Use in Facelift Surgery

Case	Indication	Protocol	Sessions	Duration of session	Time until the wound healed	Do you think HBOT improved your wound healing?
1	Delayed wound healing	Daily for 5 sessions	5	90	14	Yes, it is perfect—all patients should receive HBOT therapy following surgery
2	Enhanced wound healing	3 days in week for 10 sessions	10	60	14	I am unable to decide—nothing has changed
3	Enhanced wound healing	Daily for 10 sessions	10	90	10	Yes, there has been an improvement in health and psychological well-being
4	Enhanced wound healing	2 sessions per week	1	90	30	She is afraid of enclosed spaces and does not wish to complete the therapy
5	Enhanced wound healing	Daily for 5 sessions	5	75	7	Scars have not changed; however, bruises have improved
6	Enhanced wound healing	Daily for 6 sessions	6	60	7	Yes—improves sleep and reduces swelling in the face
7	Enhanced wound healing	Daily for 5 sessions	4	60	14	I am not able to make a decision yet, but the doctor says that the wound is healing
8	Enhanced wound healing	4-5 days a week for 10 sessions	15	90	14	Yes, plus better sleep
9	Enhanced wound healing	Daily for 10 sessions	9	90	10	Yes

## DISCUSSION

HBOT has had lots of proposed implications in the context of wound management, specifically in the scope of healing and aesthetic outcome improvement.^10^ Our study assessed the utilization of HBOT in facelift surgery. Overall, the outcomes of HBOT utilization were very favorable in our study. The duration until wounds healed in the HBOT group was significantly lower compared to the control groups. Moreover, the vast majority of the patients enrolled in our study were enrolled with a primary objective of achieving general wound healing. Studies have shown HBOT to be effective in improving wound healing in plastic surgery.^11^ Patients undergoing facial surgery desire a fast recovery so they can return to their daily activities.^12^ The mean duration required for wound healing was dramatically lower in the HBOT group compared to the control group, hence supporting the primary objective of time reduction until reaching a desired aesthetic outcome. The average number of HBOT sessions required in our study group was 7.22. A study conducted by Teguh et al^12^ showed an average of 48 sessions of HBOT required in their study population. In our study group, nearly half of the sampled individuals have some sort of underlying chronic illness. Chronic illness is among the significant factors that can alter the efficacy of HBOT. Several studies proved that despite the presence of comorbidities, HBOT utilization produced a better outcome compared to not using it.^12–15^ Subsequently, HBOT has been linked to an enhancement of quality of life following aesthetic surgery.^16^ The curative advantages of HBOT are associated with the interdependence of gas concentration, volume, and pressure.^17^ The scope of benefits derived from HBOT extends beyond its aesthetic implications. The therapeutic effects of HBOT have been well-established in numerous studies, indicating that it can enhance neovascularization, activate fibroblasts, improve the immune response, reduce inflammation, increase growth factor synthesis, improve antibiotic and antibacterial processes, augment antioxidant response, and alleviate ischemia-reperfusion injury.^17,18^

It is important to address the relevance and limitations of the patient-specific subjective questionnaire regarding HBOT. Although the questionnaire provided insights into patient acceptance and positive perceptions of the treatment, it does not directly assess the efficacy of HBOT. While positive patient perspectives suggest that many individuals would be open to undergoing HBOT if it became standard of care, it is crucial to recognize that efficacy can only be determined through rigorous clinical evaluations and objective outcome measures. Therefore, future research should focus on conducting comprehensive clinical studies to establish the efficacy of HBOT in facelift procedures, considering both objective measures and patient-reported outcomes.

Alternative methods to enhance wound healing have gained significant attention in recent years, offering promising strategies to complement conventional approaches. One such method is the use of honey as a topical agent due to its antibacterial and anti-inflammatory properties.^19^ Additionally, evidence suggests that the application of photobiomodulation therapy, involving the use of low-level laser therapy, can accelerate wound healing.^20^

### Limitations and Future Recommendations

The comparability of the cohorts in our study is a significant strength. We carefully evaluated and matched both cohorts, ensuring similarity in demographics. This rigorous process minimizes confounding variables and increases the internal validity of our findings regarding the efficacy of HBOT on facelift outcomes. The robust methodology of cohort comparability strengthens the reliability and validity of our study. Despite the promising results of this study, there are several limitations to consider. Firstly, the small sample size, which was limited to female patients, may affect the generalizability of the findings. We recommend further research to encompass a broader patient base and incorporate additional photographs at various time points.

Additionally, the study only included patients from a single surgical practice, which may limit the applicability of the findings to other settings. The retrospective design of the study may also have introduced bias or confounding variables, and the absence of blinding may have affected the results. We suggest that future research should incorporate more objective methods for assessing wound healing, as this would offer a robust and unbiased evaluation, ensuring accuracy and reliability in determining the time to wound healing. Additionally, we recommend utilizing FACE-Q as an instrument for patient-reported outcome assessment. Unfortunately, to date there is no protocol for how many sessions are needed when utilizing HBOT.^18^ Subsequently, the length of the sessions lacks global standardization and is determined by the physician.

Furthermore, there was a lack of randomization and control of other adjunctive therapies used in the control group. To build on the findings of this study, future research should use larger sample sizes with more diverse populations and a randomized controlled trial design to minimize bias and increase the generalizability of the results. Additionally, including patients who underwent different aesthetic surgery procedures may provide a more comprehensive understanding of the benefits of HBOT. One limitation of our study is that a patient in the HBOT group received only a single HBOT treatment. The impact of a single treatment on outcomes may be limited, and it is important to interpret the findings related to this patient with caution. Further research is needed to explore the optimal number and frequency of HBOT treatments and determine their meaningful effect on facelift outcomes.

Finally, evaluating the efficacy of HBOT compared to other adjunctive therapies for postoperative wound healing would provide a more complete understanding of the role of HBOT in aesthetic surgery practice. We recommend future studies consider the cost considerations and alternative adjunctive modalities, which were important aspects raised in the review. While our study primarily focused on assessing the efficacy of HBOT on facelift outcomes, we acknowledge the need to evaluate cost implications and explore alternative treatment options.

## CONCLUSIONS

HBOT is a supplemental therapy that effectively delivers oxygen systemically to maintain tissue viability during such complications. The effects of HBOT on the enhancement of aesthetic outcomes postfacelift surgery in our sample were very favorable. There was a statistically significant correlation linked to the efficacy of HBOT in markedly reducing postoperative healing time in comparison with the group that did not receive HBOT. We recommend that future studies be conducted at larger scales to assess the effect and significance of HBOT on a wider spectrum.
